# Media depictions of primary care teleconsultation safety: a thematic analysis of UK newspapers

**DOI:** 10.3399/BJGP.2023.0543

**Published:** 2024-08-06

**Authors:** Kaiyang Song, Molly Hey, Rebecca Payne

**Affiliations:** Medical Sciences Division, University of Oxford, Oxford.; Medical Sciences Division, University of Oxford, Oxford.; National Institute for Health and Care Research In-Practice Fellow, Nuffield Department of Primary Care, University of Oxford, Oxford.

**Keywords:** media analysis, patient safety, patient care, primary health care, remote consultation

## Abstract

**Background:**

The COVID-19 pandemic necessitated the widespread roll-out of teleconsultations across primary care services in the UK. The media’s depiction of remote consultations, especially regarding their safety, is not well established. These insights are important: newspapers’ coverage of healthcare-related news can influence public perception, national policy, and clinicians’ job satisfaction.

**Aim:**

To explore how the national newspapers in the UK depicted both the direct and indirect consequences of the remote-first approach on patient safety.

**Design and setting:**

We performed thematic analysis of newspaper articles that discussed patient safety in primary care teleconsultations, which were published between 21 January 2021 and 22 April 2022.

**Method:**

We identified relevant articles using the LexisNexis Academic UK database. We categorised data from these articles into codes before developing these into emergent themes through an iterative process.

**Results:**

Across the 57 articles identified, the main safety concern identified was missed and/or delayed diagnoses over tele-appointment(s), while isolated cases of inappropriate prescribing were also reported. The media reported that the transition to a remote-first approach reduced the accessibility to primary care appointments for some groups (especially patients with lower digital literacy or access) and heightened the burden on other healthcare services; in particular, there were reports of patient care being compromised across NHS emergency departments.

**Conclusion:**

The print media predominantly reported negative impacts of remote consultations on patient safety, particularly involving missed and/ or delayed diagnoses. Our work highlights the importance of further exploration into the safety of remote consultations, and the impact of erroneous media reporting on policies and policymakers.

## Introduction

Before the COVID-19 pandemic, the prevalence of remote consultations across primary care practices in the UK was on the rise.^1^ The trend towards remote consultations, which in the UK had typically entailed telephone consultations instead of video calls,^2^ was greatly accelerated by the pandemic in order to reduce transmission.^3^ Here, the term ‘remote consultation’ refers to teleconsultations (that is, consultations involving communication through electronic means, for example, telephone) whereby the patient is located remotely from the clinician with no face-to-face contact.^4^ These consultations may be conducted in real time (synchronous, that is, through phone call or Zoom platform) or through a delayed response system (also known as asynchronous or store-and-forward) through e-consult forms.^5^

In March 2020, NHS England accelerated the roll-out of a total triage model, whereby patients initially provided information over the phone or electronic forms regarding the nature of their condition or symptoms, before a decision was made regarding the type of consultation.^6^ This framework underpinned efforts to reduce footfall, and, in turn, COVID-19 transmission across primary care services; within a month, 85% of all consultations were performed remotely.^7^ As the pandemic unfolded, updated guidance from NHS England in May 2021 demanded that practices offered face-to-face (F2F) appointments and patients should have autonomy over the consultation type.^8^ Despite this directive, changes were viewed by the media as slow to manifest, culminating in the 2021 national *Daily Mail* campaign for more F2F primary care appointments.^9^

The advantages and disadvantages of remote consultations have been widely documented across the medical literature.^10^^,^^11^ While two key studies have also evaluated the merits and limitations of remote consultations, as portrayed by the UK newspaper media,^9^^,^^12^ an in-depth analysis of how the UK print media has depicted the safety of teleconsultations (particularly over an extended and continuous time frame) is currently lacking. Such work is important. Negative media coverage towards health care and medical professionals influences public perceptions and indirectly impacts patient safety,^13^^,^^14^ and contributes to GPs’ work stress and reduced job satisfaction.^15^ Media campaigns can gain a momentum that changes political priorities, leading directly to policies in conflict with scientific consensus.^16^^–^18 Examples of this have been seen in previous disease outbreaks, such as Ebola, where border screening known to be ineffective was introduced in response to public pressure.^18^

**Table:** How this fits in

While previous work has shown that UK print media articles report risks of delayed diagnosis and inappropriate prescribing in relation to remote consultations, our study explores these areas in greater depth. The analysis also highlights the inaccessibility of remote consultations for certain groups, and the increased burden that the remote-first approach placed on non-primary care healthcare services. The media drove a narrative that remote care impacted on emergency departments, despite no evidence of this happening. With remote consultations remaining commonplace as the COVID-19 pandemic settled, the media increasingly reported negative public perceptions of healthcare services and GPs; this is significant given the known impact of negative media coverage on clinicians’ wellbeing. Taken together, future work should explore if the safety concerns highlighted by media articles are accurate, as well as investigate how clinicians could be supported and encouraged to voice their concerns, amid negative media coverage.

Here, we build on previous analysis of UK newspaper articles about remote consultations, which have typically focused on the narrative techniques used by the media, attitudes towards GPs, and policy changes.^9^^,^^12^ We offer a novel perspective by analysing in depth how the print media portrays the safety of remote consultations.

## Method

This study followed the Standards for Reporting Qualitative Research,^19^ and aimed to offer a transparent, rigorous, and complete overview of the media’s portrayal of patient safety across remote consultations. The nature of our study, namely, a comprehensive analysis of print media articles, lent itself to a qualitative case-study approach.

We initially searched the eight most widely read newspapers across the UK (*The Telegraph*, *Daily Mail*, *The Times*, *The Guardian*, *Express*, *The Independent*, *The Sun*, and *Daily Mirror*), including their Sunday editions, for newspaper articles that reported specific safety incidents or stories in primary care between 21 January 2021 and 22 April 2022. We also searched *The Voice,* which describes itself as *‘Britain’s favourite Black newspaper’*. We performed our search through the LexisNexis Academic UK database (https://signin.lexisnexis.com/lnaccess/app/signin?back=https%3A%2F%2Fplus.lexis.com%3A443%2Fuk&aci=uk) and *The Voice’s* website (https://www.voice-online.co.uk) using the key search terms shown in Box 1.

**Box 1.** Search terms

Search terms: (video consultation OR video consultations OR online consultation OR online consultations OR phone consultation or phone consultations OR telephone consultation OR telephone consultations OR virtual consultation OR virtual consultations OR digital consultation OR digital consultations OR video consult OR video consults OR online consult OR online consults OR phone consult OR phone consults OR telephone consults OR virtual consult OR virtual consults OR digital consult OR digital consults OR video appointment OR video appointments OR online appointment OR online appointments OR phone appointment OR phone appointments OR virtual appointment OR virtual appointments OR digital appointment OR digital appointments OR telephone appointment OR telephone appointments OR remote consultation OR remote consultations OR remote consult OR remote consults OR remote appointment OR remote appointments OR email consultation or email consultations OR email consult OR email consults OR email appointment or email appointments or text consultation or text consultations or text consult or text consults or text appointment or text appointments) AND (GP OR GPs)

In total, 583 articles were initially identified. Articles were manually screened by a single reviewer who included news articles reporting specific patient safety incidents and excluded opinion pieces, readers’ letters, and articles summarising findings from research studies, policy reports, or ‘expert’ opinions (which may refer to safety issues second-hand). We focused on articles reporting specific patient safety incidents or stories, owing to the practicality of this approach, and because these articles were felt to be most conducive to our qualitative case-study methodology. Sixty cases reporting safety incidents were identified. Full-text review was then undertaken by two authors. Three further articles were excluded as irrelevant to the study aims, leaving 57 news articles reporting specific safety incidents in remote primary care.

We analysed the media articles using a thematic approach.^20^ The lead author reviewed all the articles twice before developing initial codes that were then grouped into emergent themes relating to patient safety. A second author also read all the articles and independently developed their own codes and themes. Both authors then underwent an iterative process to compare their respective codes and themes. Any discrepancies were discussed with a third author and, through referring back to the raw data, a consensus was reached on four themes (Box 2).

**Box 2.** Themes and corresponding sub-themes relating to patient safety, following qualitative analysis of 57 UK media articles between 21 January 2021 and 22 April 2022

**Theme**	**Sub-themes**
Impact of remote-first approach on patient safety in general practice	Inappropriate diagnoses over remote consultations
	Inappropriate prescribing over remote consultations
	Limitations of remote compared with face-to-face consultations
Impact of remote-first approach on accessibility to GP services for different groups	Decreased accessibility to services across certain groups (for example, lower digital literacy, no internet access, and language barriers)
	Increased accessibility to services across certain groups (for example, high digital literacy and patients with mobility issues)
Impact of remote-first approach in general practice on patient safety across other healthcare services	Increased burden on other community healthcare providers (for example, pharmacists)
	Increased uptake of private health care
	Increased burden on 111 and A&E
Public attitudes towards primary care and remote-first approach during the pandemic	Risk of COVID-19 transmission
	Attitudes towards remote consultations
	Attitudes towards GPs during the pandemic

*A&E = accident and emergency.*

The research team consisted of a final-year clinical medical student, a 5-year clinical medical student, and a GP academic. One author holds an interest in media narratives and has also previously conducted research regarding digital health technologies. Two authors have experience performing qualitative analysis and have conducted research into patient safety. One author has previously published qualitative work on the safety of remote primary care consultations. Although the authors were aware that general practice has been portrayed negatively by the media in the past, when conducting this study the authors aimed to mitigate any researcher bias by using a robust, transparent, and systematic approach during screening, coding analysis, and data interpretation (as outlined above).

## Results

Our search strategy identified 57 relevant articles from seven national newspapers (no relevant articles were identified from *The Guardian* or *The Voice*). Following qualitative analysis, four main themes were identified in relation to the print media’s coverage of telemedicine and the implications on patient safety. Box 2 shows the themes and corresponding sub-themes. Here, we consider each theme, focusing on the media’s depictions of the impact of remote consultations on patient care.

### Impact of remote-first approach on patient safety in general practice

The impact of the transition to remote consultations on missed or incorrect diagnoses has been widely discussed by the print media. One article quoted data from Macmillan Cancer Support, which highlighted that *‘up to 50 000 people’* (*The Telegraph*, 12 May 2021) had undiagnosed cancer during lockdown, a figure, in part attributable to remote consultations and GPs barring *‘patients from their Fort Knox surgeries’* (*The Telegraph*, 12 May 2021). Elsewhere, another article highlighted that *‘around 60 000 diagnoses of type 2 diabetes …* [were] *missed or delayed between March and December 2020’* (*Daily Mail*, 30 April 2021). Several articles referenced specific ‘prevention of future death (PFD)’ coroner reports; for example, in the case of Maurice Leech, who was aged 99 years, their fractured leg was missed over a telephone consultation, and it was reported that *‘a physical examination would probably have resulted in Mr Leech being referred back to hospital at an earlier stage’* (*Daily Mail*, 10 September 2021). In another PFD, one patient, who had a history of addiction and medication abuse, reportedly received an inappropriate pain medication prescription over teleconsultation. This example relates to the sub-theme on inappropriate prescribing over remote consultations:

*‘… a history of addiction, self-harm and poor use of prescribed and illicit substances. Prescribing of these* [pain] *medications was done through telephone consultations due to Covid-19 and on occasion additional replacement prescriptions were given with little challenge.’*

(*The Times*, 10 September 2021*)*

Many cases of ‘red flag’ symptoms were reported as not being assessed in person: *‘Patients with blood in their urine, severe ongoing stomach pain, unusual swellings under the skin and significant, unintended weight loss were all offered telephone appointments only’* (*Daily Mail*, 4 April 2021). In numerous articles, remote consultations did not appear to have identified the severity and nature of patients’ symptoms. This corresponds with the sub-theme on inappropriate diagnoses over remote consultation:

*‘*[In March 2021] *I got a phone appointment with my doctor, was told I had a urine infection and got antibiotics … In August I asked to speak to another doctor, who sent me for an ultrasound CT scan and biopsies. I was then admitted to hospital where I was told on the ward I had cancer.’*

(A patient, *The Sun*, 20 August 2021)

*‘*[Regarding David Nash, a 26-year-old who died after mastoiditis led to meningitis, but was misdiagnosed over several remote consultations, his father asked:] *how do you diagnose an ear infection … without actually looking in the ear?’* (Andrew Nash, David Nash’s father, *Express*, 18 October 2021)

Not identifying the severity and nature of patients’ symptoms reinforced the view of the Silver Voices group (campaign group for people aged >60 years) that *‘it’s inherently unsafe to rely on telephone diagnosis’* (*The Telegraph*, 6 January 2022). One prominent example was that of Joy Stokes who was aged 69 years and died after her cancer was initially mistaken over a remote consultation for arthritis. A cancer nurse told her that her condition would have been *‘controllable if only she’d got there earlier’* (*The Telegraph*, 12 May 2021).

Aside from outlining cases of misdiagnoses and/or inappropriate prescribing over remote consultations, the media also frequently highlighted clinicians’ views towards F2F consultations and teleconsultations. Across several articles, GPs reportedly outlined an important advantage of F2F appointments; namely, certain signs and conditions (for example, anaemia, melanoma, and Parkinson’s disease) could be identified by observing patients in person, relating to the sub-theme on limitations of remote compared with F2F consultations:

*‘I’ve spotted melanoma skin cancers in patients who’ve come in for other problems, and Parkinson’s in a patient just because of the way she walked into the consulting room, but the digital model removes the option of opportunistic or preventative healthcare. It treats a symptom, not the patient.’*

(A GP, *Daily Mail*, 9 May 2021)

*‘By observing someone I can tell, for example, if they are anaemic. The same goes with weight if they have lost weight, I will know that from just seeing them because I have known them for so long.’*

(A GP, *Daily Mail*, 30 April 2021)

Moreover, a separate clinician expressed that in F2F appointments, *‘there are non-verbal cues and body language that you pick up on’* (*Daily Mail*, 30 April 2021). However, the media also quoted clinicians who offered a counterargument; for example, in one article, a GP expressed that *‘remote appointments were appropriate in the majority of cases as long as doctors took a careful history, often supplemented with video calls or photos’* (*Daily Mail*, 4 April 2021). Furthermore, a survey of 1000 GPs revealed that *‘57 per cent believe the flexibility of offering remote consultations has improved care’* (*Daily Mail*, 10 September 2021).

### Impact of remote-first approach on accessibility to GP services for different groups

Print media articles commonly highlighted how the transition to a remote-first approach during the pandemic had varying effects on accessibility to GP services across different groups. Concerns about the accessibility of remote consultations were reported to be particularly prominent in populations with limited technology literacy. This relates to the sub-theme on decreased accessibility to GP services across certain groups (for example, lower digital literacy, no internet access, and language barriers).

*‘… a woman who was struggling to see out of a swollen eye was told that she would need to send photographic evidence or complete an online questionnaire. Campaigners for the elderly said pensioners were being left “frightened” and were being put at risk by a system that relied on them to be digitally savvy.’*

(*The Telegraph*, 12 October 2021)

Accessibility concerns were raised by Age UK data, which revealed that *‘almost half of over-75s — about two million Britons — are not online’* (*Daily Mail*, 9 May 2021). Concerns were also raised *‘that hearing loss can make telephone consultations challenging’* (*Th*e *Telegraph*, 21 August 2021), alongside the inaccessibility of remote consultations for people who *‘don’t have access to the internet, for whom English is not their first language or those in a mental health crisis’* (Jacob Lant, head of policy at Healthwatch England [at the time of reporting], *The Times,* 10 September 2021). Altogether, these factors have led to the suggestion in one article that *‘serious conditions are going undiagnosed because so many people … don’t feel comfortable or able to have remote consultations’* (Dennis Reed, director of Silver Voices, *The Telegraph*, 21 August 2021), reinforcing the findings regarding missed diagnoses and/or inappropriate prescribing.

Conversely, some patients, especially younger individuals and/or patients with underlying mobility problems, have reportedly found the remote-first approach to have improved access to GP services, illustrating the sub-theme on increased accessibility to GP services across certain groups (for example, high digital literacy and patients with mobility problems):

*‘We know that many patients have benefited from receiving care remotely, and as a result found access to our services easier and more convenient, particularly for patients with mobility problems and younger people.’*

(Martin Marshall, chair of Royal College of General Practitioners [at the time of reporting], *The Times,* 18 September 2021).

Patients with high digital literacy and those with mobility problems *‘like the convenience of the new* [remote-first] *system, and more are able to get a same-day appointment, albeit remotely’* (*Daily Mail*, 4 April 2021). Interestingly, results from the publication *Pulse* on its poll of GPs suggested that the remote-first approach helped to relieve waiting times: *‘Patients are securing in-person consultations quicker than before the pandemic, with waits cut from 15 days in August 2019 to nine now’ (Daily Mail*, 10 September 2021).

### Impact of remote-first approach in general practice on patient safety across other healthcare services

From 2021–2022, the UK print media heavily reported that the declining availability of F2F primary care consultations culminated in patients opting for alternative healthcare services, including community healthcare providers. This relates to the sub-theme on increased burden on other community healthcare providers (for example, pharmacists):

*‘*[Because of the unavailability of GP appointments, the local pharmacist at Hightown has become the] *de facto GP, and his consulting room a de facto surgery …* [he has] *been dealing with all manner of serious conditions and emergencies …* [including] *patching people involved in cycling accidents, people with lacerations.’*

(*Daily Mail*, 4 September 2021)

It was reported that patients also opted for private healthcare options, which corresponds to the sub-theme on increased uptake of private health care:

*‘… wife had previously been diagnosed with the blood cancer chronic lymphocytic leukaemia, was told to try throat lozenges during a telephone consultation when she complained of breathlessness and problems swallowing …* [private] *consultant immediately admitted her to hospital and diagnosed her with an aggressive non-Hodgkin lymphoma, which had spread to her lungs, and sepsis.’*

(*Daily Mail*, 9 May 2021)

In addition, the reporting also stated that patients had to turn to other NHS services, relating to the sub-theme on patients presenting to 111 and accident and emergency (A&E), often with a delayed presentation:

*‘Her GP … refused face-to-face appointments, misdiagnosed her with irritable bowel syndrome and prescribed medication for depression …* [a couple months later, she] *was rushed to A&E after suffering severe bleeding. A CT scan revealed … stage four bowel cancer.’*

(Regarding a patient who had developed stomach cramps and was losing weight, *Daily Mail*, 9 October 2021)

Most notably, A&E services across the UK were reported to bear the biggest brunt, with one article highlighting the results of an NHS survey: *‘nearly one in ten who couldn’t see a GP attended A&E instead’* (*Daily Mail*, 9 October 2021). Accordingly, many patients were reported as losing faith in the accessibility of GPs: *‘I don’t bother* [taking his unwell husband to the GP] *anymore — I just take him to A&E’* (*Daily Mail*, 9 May 2021). A sentiment echoed by Dennis Reed, the director of Silver Voices: *‘if you go to A&E, you may have to wait for four or five hours, but at least you will be seen that day’* (*Daily Mail*, 9 October 2021).

The media frequently highlighted cases of patients presenting to A&E inappropriately, with insufficient clinical indication. One A&E clinician reportedly stated, *‘I’m seeing patients with trivial things like an ankle complaint or an unusual discharge … these are conditions that aren’t appropriate for A&E and can be easily dealt with by* [general] *practices’* (*Daily Mail*, 4 April 2021). These changes in practice appeared to concern the Royal College of Emergency Medicine (RCEM). In one article, both the lack of access to primary care and the shift to virtual consultations were viewed by the RCEM as contributing to *‘dangerous crowding in A&Es which is unsafe and unconscionable and threatens patient safety*’ (*Daily Mail*, 9 October 2021).

### Public attitudes towards primary care and remote-first approach during the pandemic

While the initial total triage approach and shift towards remote consultation was reportedly welcomed by the public as a *‘sensible precaution at the height of the pandemic’* (*Daily Mail*, 30 April 2021), this sentiment waned as the UK saw *‘falling Covid case rates and the vaccination of health workers’* (*Daily Mail*, 4 April 2021)*.* Towards mid-to-late 2021, many patients and journalists alike expressed concerns that COVID-19 was used as *‘an excuse*’ (*Express*, 24 January 2021) and *‘cover for driving through a change in working practices’* (*The Telegraph*, 5 May 2021).

In one article, the mother of one patient with cancer highlighted that *‘dealing with the real risk of Covid should not create a higher risk of cancer death’* (*Daily Mail*, 15 September 2021).

Across other articles, members of the public reportedly questioned why they could not see a GP in person, despite national COVID-19 restrictions being lifted. This relates to the sub-theme on risk of COVID-19 transmission:

*‘I have been to my dentist and had my teeth checked. I go to a supermarket with a mask and buy my weekly food, and have even been to my solicitors and had a face-to-face consultation with masks. I have been to A&E because of the excruciating pain I am in. So why can’t I see my GP?’*

(A patient, *Daily Mail*, 4 April 2021)

The public also repeatedly expressed their preference for F2F appointments, illustrating the sub-theme on attitudes towards remote consultation:

*‘I’ve got issues I can’t get answered with the doctor because I like to see people face-to-face, I have no confidence when I talk to somebody over the phone.’*

(A patient, *The Telegraph*, 14 May 2021)

*‘I do find it very hard to speak to my GP on the phone, as I feel I’m taking up their time and can’t express what I really feel. I’m feeling very sad and not wanted any more. I’d rather not bother anyone.’*

(A patient, *Daily Mail*, 4 April 2021)

*‘Roughly half of respondents felt their care or experience was not as good* [with remote consultations] *as it would have been otherwise.’*

(Results from a Patients Association report, *Daily Mail*, 30 April 2021)

The delayed return to F2F appointments, as evidenced by data from one article, *‘Before the pandemic, 80 per cent were face-to-face but now* [October 2021] *it is now just 58 per cent’* (*The Times*, 12 September 2021), appeared to propel negative attitudes towards GPs. This corresponds to the sub-theme on attitudes towards GPs during the pandemic:

*‘… the public perception of GPs is that they are overpaid — the average GP wage now tops £100,000 — and underworked, dragging their feet in getting back to the surgery to resume a normal service.’*

(A patient, *Daily Mail*, 4 September 2021)

## Discussion

### Summary

The introduction of remote consultations in primary care during the COVID-19 pandemic received largely negative coverage by the UK print media, with well-documented concerns regarding patient safety.

From 2021–2022, the UK media commonly portrayed the negative impact of remote consultations on patient safety, most commonly highlighting instances of missed or delayed diagnoses. Concerns surrounding the indirect impact on patient safety across other NHS services, especially emergency departments, appear to have been largely unfounded. It is important to highlight that these media articles referred to a small selection of specific cases, in the context of millions of consultations that would have occurred in the same time frame.

### Strengths and limitations

There are several notable limitations of our study. First, newspaper articles are unlikely to provide a representative portrayal of primary care practices across the UK during the pandemic. Previous work has shown the frequent bias of media articles towards negative headlines with a focus on specific anecdotes or cases.^21^^,^^22^ This is especially pertinent in our study: 36 out of the 57 articles analysed were published by either the *Daily Mail* or *The Telegraph,* which are two national newspapers that spearheaded public campaigns against remote consultations.^9^ Second, despite our thorough search of the extensive LexisNexis Academic UK database, it is feasible that some articles were missed. Third, our search strategy was limited to articles up to April 2022; future work is required to establish whether remote consultation practices and the corresponding media portrayals changed as society returned to pre-pandemic living. Furthermore, our search was limited to articles reporting specific stories or incidents relating to patient safety and teleconsultations; future work should encompass analysis of other article types, including expert or opinion articles.

More significantly, our study solely focused on print media articles; we did not explore whether the rhetoric of newspaper articles was mirrored across alternative media platforms (for example, radio, television, or social media). This is particularly relevant given that, relative to print media, these alternative media forms are gaining popularity, and have ever-growing potential to shape public perception and policy.^23^^,^^24^ Thus, there is a need for future studies to explore how the safety of remote consultations has been explored across alternative media platforms. Such work could employ a similar case-study qualitative approach and could involve searching readily available online archives of previous TV and/or radio broadcasts and transcripts.

### Comparison with existing literature

Our study extends the preliminary work done in this area by Mroz *et al*, who identified that the UK media has frequently reported cases of missed or delayed diagnoses, and inappropriate prescribing during remote consultations over two fortnightly periods (13–26 May 2021 and 14–27 October 2021).^9^ By focusing on newspaper articles across a continuous and wider period, and by concentrating specifically on safety, we offer novel insights. First, we show that specific reported concerns regarding remote consultations, compared with F2F appointments, included difficulties for clinicians to interpret patients’ body language, as well as to identify certain signs or conditions remotely (for example, anaemia, melanoma, and Parkinson’s disease). Second, the print media frequently highlighted the inaccessibility of remote consultations for specific groups (for example, older patients, patients with language barriers, and patients with reduced digital literacy). Furthermore, the remote-first policy reportedly compromised patient safety and care across other healthcare services; notably, the media documented an overwhelming influx of patients self-presenting to NHS emergency departments.

There are several reasons why it is important to consider the UK media’s narrative towards health care and healthcare professionals. First, prior work has shown that newspaper portrayals of general practice are largely negative; ongoing ‘GP bashing’ is viewed as a contributory factor for clinicians’ decisions to leave the profession.^25^^,^^26^ Second, media rhetoric has been shown to shape public perceptions towards healthcare issues and medical professionals, with well-documented cases of stories leading to public misperceptions.^13^^,^^14^ Finally, previous studies have outlined that, on occasion, media campaigns can influence public policies.^27^^,^^28^

The UK media frequently highlighted the value of in-person consultations for allowing physical examinations and assessment of patients’ overall wellbeing. This is a view supported by the literature. In one survey of primary care physicians across six states in the US, the inability to conduct a physical examination was viewed as the biggest challenge of remote consultations.^29^ Elsewhere, 64% of primary care clinicians stated they would not be confident to diagnose a patient over telemedicine.^30^ Other studies have highlighted that remote consultations lead to reduced appreciation of the content and tone of patients’ dialogue, and a curtailed patient–doctor therapeutic relationship.^31^^,^^32^ Accordingly, a study spanning 18 general practices in Scotland found that clinicians are less likely to acquire adequate information that helps to safely include or exclude relevant diagnoses across remote consultations.^32^ In spite of these data, it is important to outline that clinicians and patients frequently report high levels of satisfaction with teleconsultations;^32^^,^^33^ they offer patients increased convenience and accessibility to health care, while improving appointment adherence.^11^

Regarding the impact of remote medicine on patient care, there is some evidence to suggest that teleconsultations do not compromise treatment efficacy or patient safety.^34^^,^^35^ However, these studies are limited by their focus on medically stable patients with chronic conditions (for example, diabetes and hypertension) and/ or on patients who self-select or are selected by clinicians to have remote consultations.^35^^,^^36^ It remains largely unexplored whether remote consultations have been associated with significant increases in rates of misdiagnoses and/or other threats to patient safety across the general population during the COVID-19 pandemic.

Another facet of patient safety discussed in the media was the burdens on other healthcare services, especially emergency departments. This view was echoed by the health secretary giving oral evidence to the Health and Social Care Committee.^37^ Although this was a concern heavily raised by media reports, previous intra-pandemic studies have shown that remote consultations are not significantly associated with admission to emergency departments or hospitalisations.^35^^,^^38^ Moreover, a UK study showed a reduction in adult patients attending emergency or acute medicine departments during the first 2 years of the pandemic.^39^

This disparity with our media analysis may be explained by the tendency of media articles towards isolated cases and anecdotal experiences, which, in turn, may have led to a misrepresentation of the clinical landscape at the time.^40^ The propagation of such media myths by the health secretary shows the impact that media reporting can have on policymakers keen to maintain popularity.

Our analysis also highlighted how the media correctly identified that the suitability of remote consultations may vary across patients with different demographic, digital literacy, and health characteristics. The current medical literature supports our findings that patients with lower digital literacy and/ or no internet access, and for whom there was a language barrier, often had difficulties engaging in remote consultations.^11^^,^^41^ Moreover, others have shown that teleconsultations may not be suitable for patients with cognitive or sensory impairment, and those experiencing socioeconomic deprivation.^11^^,^^41^ Furthermore, numerous studies have highlighted that teleconsultations are less appropriate for patients requiring physical examination, presenting with ‘red flag’ symptoms, or when a therapeutic relationship has yet to be established.^27^^,^^42^ Conversely, remote appointments may be appropriate for consultations involving medically stable patients with chronic illnesses, medication reviews, or discussing blood test results.^43^ Thus, to minimise the occurrence of risks to patient safety, decisions surrounding the type of consultation should be a culmination of patients’ preferences and clinicians’ judgement. The latter should consider factors including, but not limited to, the patient’s digital literacy, the purpose of the consultation, and the patient’s clinical condition, as well as the potential risks to the patient from attending an in-person consultation.

### Implications for research and practice

Little is known on the extent to which the transition to a remote-first approach may have compromised patient safety or about how these risks should be balanced against the risks of in-person consultation during the pre-vaccination era of the pandemic. Future research is needed on the impact of remote consultations on missed or delayed diagnoses, and on the impact of misrepresentative media reporting on politicians and policymakers.

## Data

The dataset is available on request to the corresponding author.
